# Protective Effect of Dexmedetomidine on Acute Lung Injury *via* the Upregulation of Tumour Necrosis Factor-α-Induced Protein-8-like 2 in Septic Mice

**DOI:** 10.1007/s10753-019-01169-w

**Published:** 2020-01-11

**Authors:** Qian Kong, Xiaojing Wu, Zhen Qiu, Qin Huang, Zhongyuan Xia, Xuemin Song

**Affiliations:** 1 Department of Anesthesiology, Renmin Hospital of Wuhan University, Wuhan, 430060 Hubei China; 2 Department of Anesthesiology and Critical Care Medicine, Zhongnan Hospital of Wuhan University, Wuhan, 430071 Hubei China

**Keywords:** acute lung injury, TIPE2, dexmedetomidine, apoptosis, inflammation

## Abstract

The aim of the present study was to investigate whether TIPE2 participates in the protective actions of dexmedetomidine (DEX) in a mouse model of sepsis-induced acute lung injury (ALI). We administered TIPE2 adeno-associated virus (AAV-TIPE2) intratracheally into the lungs of mice. Control mice were infected with an adeno-associated virus expressing no transgene. Three weeks later, an animal model of caecal ligation-perforation (CLP)-induced sepsis was established. DEX was administered intravenously 30 min after CLP. Twenty-four hours after sepsis, lung injury was assayed by lung histology, the ratio of polymorphonuclear leukocytes (PMNs) to total cells in the bronchoalveolar lavage fluid (BALF), myeloperoxidase (MPO) activity, BALF protein content and the lung wet-to-dry (W/D) weight ratio. Proinflammatory factor levels in the BALF of mice were measured. The protein expression levels in lung tissues were analysed by Western blotting. The results showed that DEX treatment markedly mitigated sepsis-induced lung injury, which was characterized by the deterioration of histopathology, histologic scores, the W/D weight ratio and total protein levels in the BALF. Moreover, DEX markedly attenuated sepsis-induced lung inflammation, as evidenced by the decrease in the number of PMNs in the BALF, lung MPO activity and proinflammatory cytokines in the BALF. In addition, DEX dramatically prevented sepsis-induced pulmonary cell apoptosis in mice, as reflected by decreases in the number of TUNEL-positive cells, the protein expression of cleaved caspase-9 and cleaved caspase 3 and the Bax/Bcl-2 ratio. In addition, evaluation of protein expression showed that DEX blocked sepsis-activated JNK phosphorylation and NF-κB p65 nuclear translocation. Similar results were also observed in the TIPE2 overexpression group. Our study demonstrated that DEX inhibits acute inflammation and apoptosis in a murine model of sepsis-stimulated ALI *via* the upregulation of TIPE2 and the suppression of the activation of the NF-κB and JNK signalling pathways.

## INTRODUCTION

Sepsis is a systemic inflammation condition triggered by severe infection and trauma [1]. Due to pulmonary susceptibility, respiratory dysfunction, such as acute lung injury (ALI) and acute respiratory distress syndrome (ARDS), is one of the most common and severe complications of sepsis [2]. ALI is characterized by increased permeability of the alveolar-capillary barrier, interstitial edema, neutrophil recruitment and inflammatory stress-induced cell apoptosis [3, 4]. The pathophysiological mechanisms of sepsis-induced ALI involve cell inflammation, cytokine production and abnormal apoptosis [5, 6]. To date, there is no effective pharmacological approach to treat ALI.

Apoptosis, known as programmed cell death, is essential for the selective elimination of cells. However, the dysregulation of apoptosis pathways has been shown to contribute to epithelial and endothelial injury, which is characteristic of ALI [7]. Apoptosis might play a vital role in regulating pro- and anti-inflammatory processes [8], and the apoptotic process is regulated by several intracellular signalling pathways, including the JNK pathway [9, 10].

Dexmedetomidine (DEX), which is a potent α-2 adrenoreceptor agonist, is widely applied in clinical conditions such as sedation, analgesia and anxiolysis. Recently, studies have shown that DEX has potent anti-inflammatory, antioxidant and anti-apoptotic effects [11–13]. Animal studies have suggested that DEX plays a protective role in a number of lung diseases, including ALI [14–16].

Tumour necrosis factor-α-induced protein-8 (TNFAIP8)-like 2 (TIPE2), which is an essential negative regulator of TLR and TCR function, has been approved to inhibit caspase-mediated apoptosis [17]. TIPE2 has been reported to be a negative regulator of the activating protein (AP)-1, NF-κB, JNK and p38 pathways [18], which are important pathways for regulating apoptosis [9, 10].

The current study was designed to test the hypothesis that DEX attenuates LPS-induced ALI through the inhibition of lung inflammation and apoptosis *via* the promotion of TIPE2 expression and the inhibition of the NF-κB and JNK pathways, which might indicate its potential application in lung injury therapy.

## MATERIAL AND METHODS

### Animals

Adult male BABL/c mice (6–8 weeks, weighing 20 to 25 g) were purchased from the Wuhan Institute of Biological Products Co., Ltd. (Wuhan, China). The mice were maintained under specific pathogen-free(SPF) conditions that provide relative humidity ranging between 55 and 65%, temperature of 22 ± 2 °C, a 12:12 h light-dark cycle, with laboratory diet and water *ad libitum*. The animals underwent an acclimatization period of 7 days before the experiment. All experiments were performed in accordance with international and institutional guidelines for animal care, and the study was approved by Medical Ethics Committee of Renmin Hospital of Wuhan University.

### Adenovirus Gene Delivery

The recombinant adeno-associated virus containing the mouse TIPE2 gene was purchased from Hanheng Company (Hanheng Biotechnology Co., Ltd., Shanghai, China). The adeno-associated virus expressing no transgene was used as negative control (pAAV-IRES-ZsGreen). Twenty-one days before CLP model construction, BALB/c mice were anaesthetized using sodium pentobarbital, and given 5 × 10^10^ vector genomes (vg) of rAAV6-FLAG-mTIPE2 (5 × 10^12^ vg/ml) in 50 μl PBS *via* intratracheally (i.t.) administration, to induce TIPE2 over-expression in the lung. Control mice were treated with control adeno-associated virus. The efficacy of the fusion protein was evaluated by Western blotting.

#### Experimental Design

Mice were randomly divided into the following groups: (1) sham group, (2) CLP group, (3) AAV-TIPE2 (TIPE2) + sham group, (4) TIPE2 + CLP group, (5) CLP + DEX and (6) TIPE2 + CLP + DEX group.

The surgical procedure to generate CLP-induced sepsis was performed on BALB/c mice. After the mice were anaesthetized with 2% sevoflurane, a middle incision (1.5 cm) in the lower quadrants of the abdomen was made. The cecum was exposed and slightly taken out of the incision. The distal three-fourths (between the colon root and cecum terminal) of the cecum was ligated with 4–0 silk suture, and subsequently punctured with a 21-gauge needle. We squeezed a little feces through the puncture wound. Then, the cecum was repositioned, and the abdominal incision was closed with sterile suture. Sham-operated control animals underwent the same procedure except for ligation and puncture of the cecum. Immediately after the surgery, the mice were intraperitoneally injected Dex (50 μg/kg) or the same volume (200 μl) of vehicles PBS. After that, the mice were injected subcutaneously with 1 ml of sterile saline for resuscitation and put into an incubator until they recovered from the anaesthesia [19, 20].

First, Kaplan-Meier survival analysis was conducted every 24 h for a total of 7 days after CLP operation. Second, at 24 h after CLP/sham modelling, animals were sacrificed by excessive chloral hydrate, bronchoalveolar lavage fluid (BALF), arterial blood and the lung tissues without lavage were collected for further studies. Lung tissues were snap-frozen in liquid nitrogen and stored at − 80 °C for later analysis. In these experiments, the number of mice was 8 per group for tissue analysis and 20 per group for survival analysis.

### Histopathological Lung Examination

Lung tissues were harvested for observing morphologic alterations at 24 h after CLP/sham modelling. The right lung lobes were dissected, washed and fixed with 4% (*v*/*v*) paraformaldehyde for 24 h at 4 °C. Lung tissues were embedded in paraffin, sectioned at 4 μm thickness, dewaxed and rehydrated and stained with hematoxylin and eosin (H&E) solution (hematoxylin, MHS16; eosin, HT110132; Sigma-Aldrich, USA) for histological examination. The stained slides were then observed with the light microscope and the digital micrographs were taken for analyzing. Histologic changes were evaluated by two independent pathologists blinded to the experiment. The histologic injury scores [21] were calculated according to the sum of the score for alveolar edema, alveolar hemorrhage, pulmonary interstitial thickening and neutrophil infiltration. Each histological characteristic was evaluated on a scale from 0 to 3: 0, normal (no injury); 1, minimal (injury to 25% of the field); 2, mild (injury between 25 and 50% of the field); 3, moderate (injury between 50 and 75% of the field); 4, severe (injury over 75% of the field).

### TdT-Mediated dUTP Nick End Labeling Staining

Apoptosis was detected and quantified with the TUNEL assay using the *In Situ* Cell Death Detection Kit (Roche Diagnostics) according to the manufacturer’s protocol. Apoptotic cells were manifested brownish staining in the cell nuclei. Ten random sections of the lung from each mice without knowledge of the group of mice from which the lung tissue was taken, and the apoptosis index was expressed as a percentage of TUNEL-positive cells. The examination was performed by two pathologists blinded to the experiment.

### Inflammatory Cell Counting and Protein Concentration in BALF

To obtain the BALF, the lungs were lavaged three times with ice-cold PBS (0.5 ml) and withdrawn each time using a tracheal cannula (a total volume of 1.5 ml). The collected BALF was centrifuged at 3000×*g* for 10 min at 4 °C and the supernatant was collected and frozen at − 80 °C for subsequent assays. The cell pellet was resuspended in PBS, and after excluding dead cells by trypan blue staining, the total inflammatory cells in BALF were determined by counting cells with a hemocytometer (Beckman Coulter, Inc). In order to analyze differential cell counting, 100 μl of BALF was centrifuged onto slides by a Cytospin (Thermo Fisher Scientific, Waltham, USA). After the slides were dried, the cells were fixed and stained using Wright Stain solution (32857, Sigma-Aldrich, USA) according to the manufacturer’s instructions. The number of polymorphonuclear neutrophils (PMNs) was classified by a laboratory technologist blinded to the experiment, to obtain the percentage of neutrophils. The frozen BALF supernatant was thawed and thoroughly mixed; then, total protein concentration was determined by BCA (bicinchoninic acid) method.

### Arterial Blood Gas Analysis

After mice were anaesthetized, the arterial blood sample was collected with a heparinized syringe from the carotid artery. The arterial blood samples were immediately injected into an ABL700 Radiometer (Radiometer America, USA) to measure pH value, partial gas pressures of oxygen (PaO_2_), PaO_2_/FiO_2_ and partial gas pressures of carbon dioxide (PaCO_2_).

### Lung Wet/Dry Weight Ratio

Lungs were excised 24 h after CLP surgery. The magnitude of pulmonary edema was determined by calculating lung wet/dry weight ratio. The left lobe of the lungs were excised, washed with PBS, blotted and then weighed to obtain the “wet” weight. The lungs were then placed in an oven for 48 h at 65 °C and weighed to obtain the “dry” weight. The wet/dry weight ratio was calculated to quantify the degree of pulmonary edema.

### MPO Activity Assay

RIPA lysis buffer was used for lysing lung tissues, and 10-mg tissue samples were taken for each test sample. After washed with cold PBS, the tissues were resuspended in 4 volumes of MPO assay buffer, and then centrifuged (13,000×*g* for 10 min, 4 °C). The supernatant was collected and transferred to clean tubes which were placed on the ice. The MPO activity was assayed using Myeloperoxidase Activity Assay Kit (Abcam, ab105136), by measuring the absorbance of the sample at 460 nm using a microplate reader (Bio-Rad Laboratories, Hercules, CA, USA). The specific MPO activity in the lungs was expressed as unit/mg protein.

### Measurement of Pro-inflammatory Cytokines in BALF

Twenty-four hours after CLP surgery, BALF was collected. The levels of TNF-α, IL-6 and IL-1β in BALF were determined using enzyme-linked immunosorbent assay (ELISA) kits according to the manufacturer’s instructions (R&D, Inc., Minneapolis, USA). The absorbance was measured at 450 nm using an ELISA reader (BioTek Instruments, Inc., USA).

### Western Blot Analysis

Twenty-four hours after CLP surgery, the lung tissues were harvested and snap-frozen in liquid nitrogen until homogenization. The lung tissues were homogenized using a homogenizer with tissue nuclear and cytoplasmic extraction reagents (Sigma-Aldrich, USA), according to the manufacturer’s instructions. Protein concentrations were determined using the BCA protein assay kit (Invitrogen; Thermo Scientific). Protein extracts (50 μg per lane) were run on sodium dodecyl sulphate-polyacrylamide gel electrophoresis (SDS-PAGE) and transferred onto polyvinylidene difluoride membranes. The resulting membranes were blocked by incubation with 5% skim milk in TBST at room temperature for 2 h on a rotary shaker, followed by washing with TBST. Subsequently, the membranes were incubated with specific primary antibody overnight at 4 °C. For immunoblotting, rabbit anti-mice-Bax (1:1000; Abcam, Cambridge, MA), caspase 3 (1:1000; Abcam, Cambridge, MA), TIPE2 (1:200; Abcam, Cambridge, MA) and Lamin A (1:1000; Abcam, Cambridge, MA), β-actin (1:1000; Cell Signaling Technology, Boston, MA), JNK and p-JNK (1:1000; Cell Signaling Technology, Boston, MA), Bcl-2 (1:1000; ProteintechGroup, Inc., Wuhan, China), NF-κB p65 (1:1000; ProteintechGroup, Inc., Wuhan, China), caspase 9 (1:500; ProteintechGroup, Inc., Wuhan, China) antibodies were used. HRP-conjugated secondary antibody was obtained from Boster Biological Technology Co. Ltd. (Wuhan, China) and diluted at 1:5000. The membranes were washed with TBST followed by incubation with horseradish peroxidase (HRP)-conjugated secondary antibody at room temperature for 1 h. The blots were washed TBST and detected using an enhanced chemiluminescence (ECL) Western blotting detection kit. The protein bands were observed using an ECL Western blotting analysis system (Bio-Rad Laboratories, Inc., USA) and quantified by densitometry (Image Lab software version 5.2.1).

### Statistical Analysis

The data are expressed as the means ± SEM. The statistical analysis was performed using GraphPad PRISM (version 7.0; GraphPad Software, Inc., La Jolla, CA, USA) by a one-way analysis of variance (ANOVA) followed by Dunnet’s least significant difference post hoc tests. Kaplan-Meier survival analysis and the log-rank test were used to analyze the survival rate. *p* < 0.05 was considered statistically significant.

## RESULTS

### Survival for 7 Days After CLP Surgery and Histological Findings

**Fig. 1:**
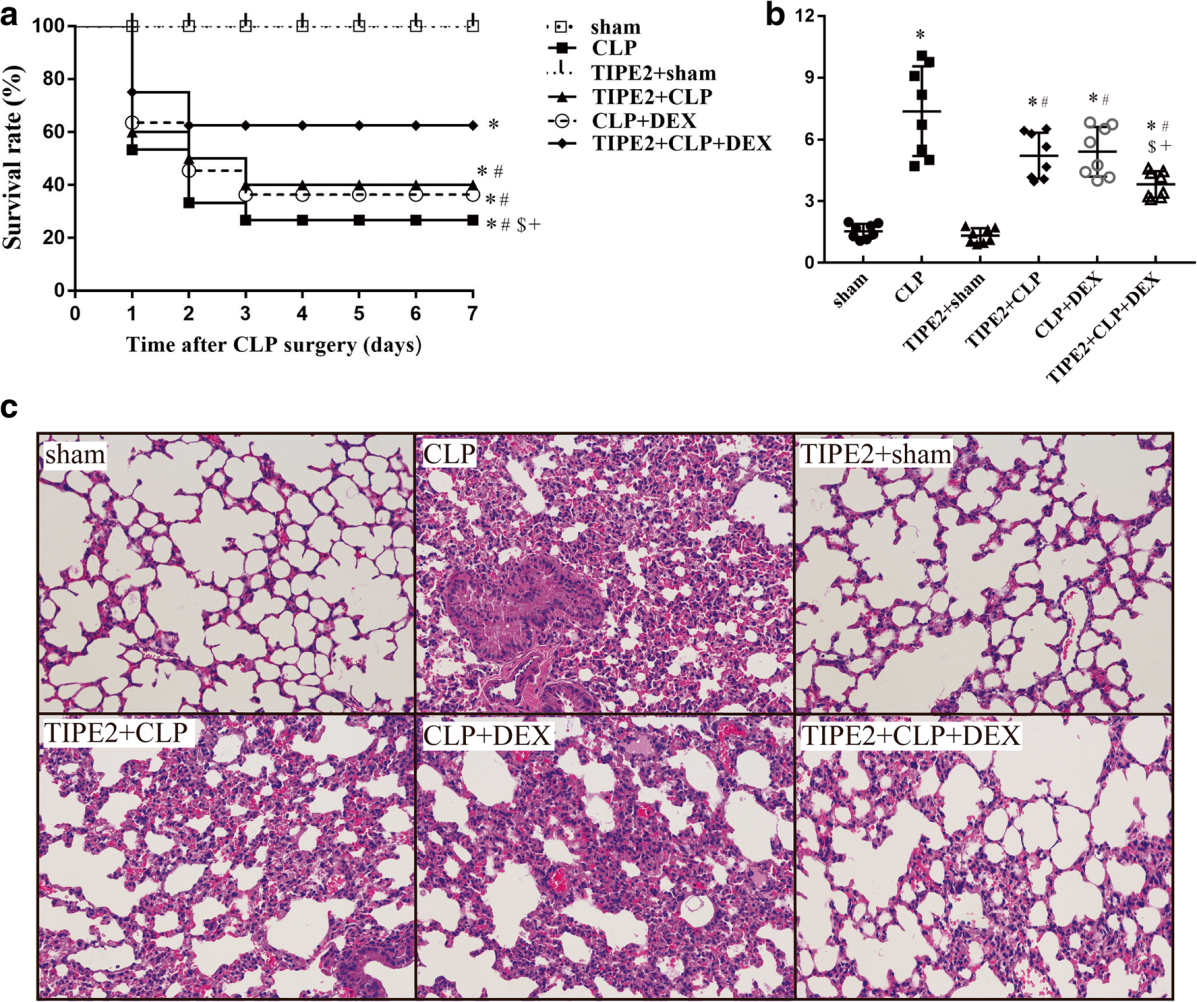
Survival for 7 days after CLP surgery and histologic changes in the lung. **a** The Kaplan-Meier survival curve shows survival for 7 days after CLP surgery. **b** Lung histologic injury score. **c** Representative histology sections of lung tissues under light microscope (H&E staining, magnification, × 200). The data are presented as means ± SEM. *n* = 8/group (*n* = 20/group in Fig. 1a), ^*^P < 0.05 *versus* sham group; ^#^*P* < 0.05 *versus* CLP group; ^$^*P* < 0.05 *versus* TIPE2 + CLP group; +*P* < 0.05 *versus* CLP + DEX group.

In the present study, we investigated the effects of DEX and TIPE2 overexpression on ALI mortality and lung histopathology in mice after the establishment of a model of CLP (Fig. 1). The survival rate post-surgery was assessed every 24 h for a total of 7 days. As shown in Fig. 1a, no mortality occurred in the sham or TIPE2 + sham groups, but CLP led to a significant decrease in the survival rate within 7 days (*P* < 0.05 *vs.* the sham group, *n* = 20/group). Lung tissues were harvested 24 h after CLP surgery and subjected to H&E staining (Fig. 1c). There were no obvious histological changes in lung tissues from the mice in the sham group. Significant pathological changes, including pulmonary capillary congestion, pulmonary interstitial edema, mass inflammatory cell infiltration into the alveolar space and lung interstitium and alveolar wall thickening, were observed in the lung tissues of mice after CLP surgery. In addition, a scoring system was used to assess the degree of lung injury. As shown in Fig. 1b, the quantitative scores of histological lung injury in the ALI mice were markedly increased compared with those in the sham group 24 h after CLP surgery. Western blotting further demonstrated that TIPE2 protein levels were significantly increased in the lung tissues of AAV-TIPE2-infected mice. Treatment with AAV-TIPE2 and DEX significantly improved the survival rate (*P* < 0.05 *versus* the CLP group, *n* = 20/group), attenuated the pulmonary histopathological changes and decreased the lung injury scores induced by CLP (*P* < 0.05 *versus* the CLP group, *n* = 8/group). Furthermore, the combination of AAV-TIPE2 and DEX had a more significant protective effect in mice that underwent CLP.

### Pulmonary Cell Apoptosis

**Fig. 2:**
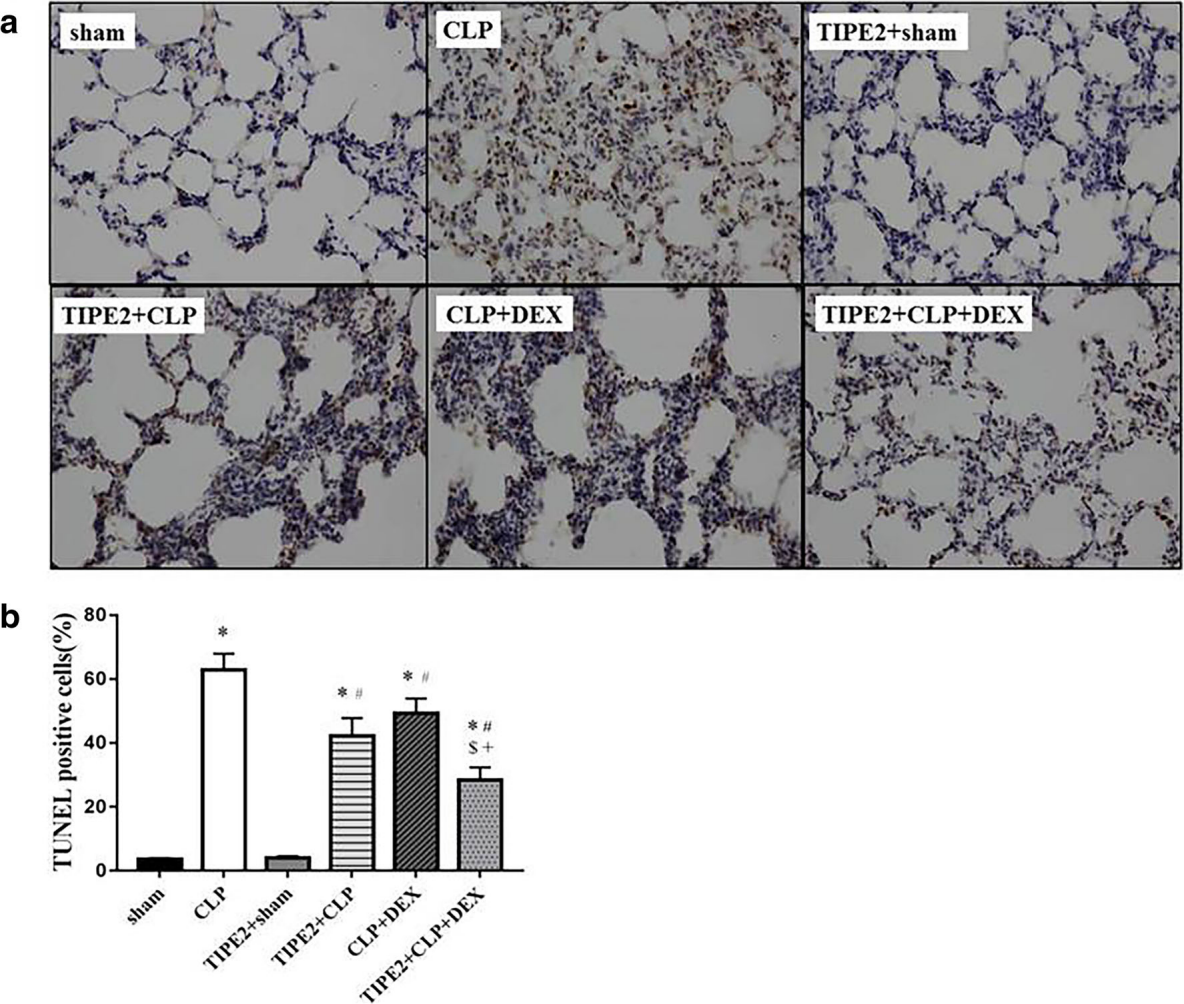
Apoptosis in the lung assessed by TUNEL assay. The lung samples were collected for measuring TUNEL staining at 24 h after CLP surgery. **a** Representative lung TUNEL staining (× 400). **b** Percentage of TUNEL-positive cells. The data are presented as means ± SEM. *n* = 8/group, ^*^P < 0.05 *versus* sham group; ^#^*P* < 0.05 *versus* CLP group; ^$^*P* < 0.05 *versus* TIPE2 + CLP group; +*P* < 0.05 *versus* CLP + DEX group.

We investigated the effects of DEX and AAV-TIPE2 on lung cell apoptosis in ALI mice by TUNEL staining. TUNEL staining revealed few apoptotic cells in the lungs of the sham group. Twenty-four hours after CLP surgery, compared with sham surgery, numerous lung cells were strongly positive for TUNEL staining. However, in the lung tissues of DEX- and AAV-TIPE2-treated mice, a few of the lung cells were TUNEL-positive (Fig. 2a). The quantitative results (Fig. 2b) showed that mice that underwent CLP exhibited a significant increase in the number of apoptotic cells (*P* < 0.05 *versus* the sham group), which was reduced by AAV-TIPE2 and DEX treatment (*P* < 0.05). Additionally, the combination treatment group exhibited fewer apoptotic cells than the AAV-TIPE2 + CLP and CLP + DEX groups.

### Pulmonary Vascular Permeability and Neutrophil Infiltration into the Lungs During ALI Induced by CLP

**Fig. 3:**
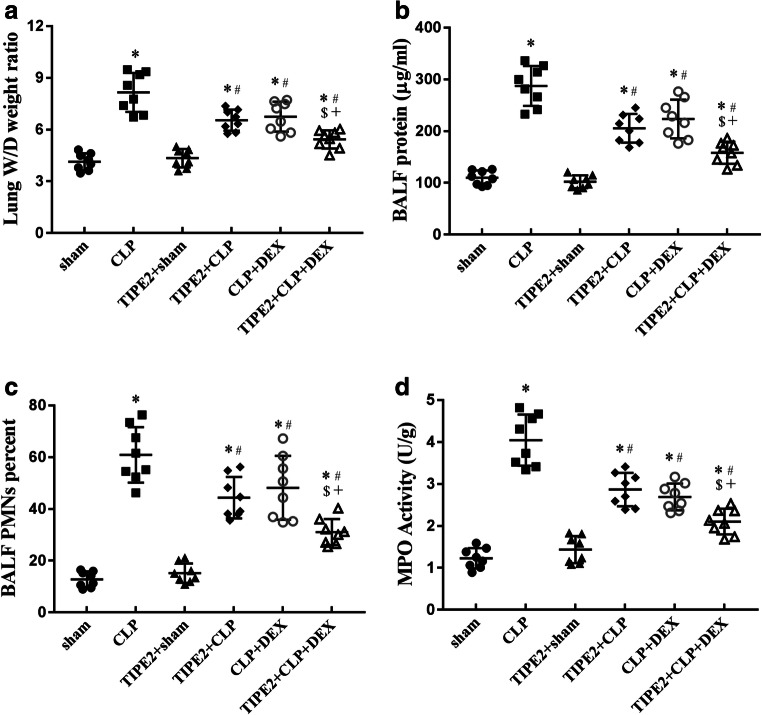
Pulmonary vascular permeability and neutrophils infiltration into the lungs during ALI. **a** Lung W/D ratio. **b** BALF protein concentration. **c** PMNs/total cells in BALF. **d** Lung MPO activity. The data are presented as means ± SEM, *n* = 8/group, ^*^P < 0.05 *versus* sham group; ^#^*P* < 0.05 *versus* CLP group; ^$^*P* < 0.05 *versus* TIPE2 + CLP group; +*P* < 0.05 *versus* CLP + DEX group.

The W/D weight ratio of the lungs and BALF protein concentration are two commonly used indicators of pulmonary vascular permeability, which is an important characteristic of ALI/ARDS. CLP-challenged mice showed a significant increase in the lung W/D weight ratio (Fig. 3a) and BALF protein (Fig. 3b) concentration when compared with those of the sham group, and these indicators were decreased by AAV-TIPE2, DEX and AAV-TIPE2 + DEX treatment (*P* < 0.05, *n* = 8/group). We also detected the ratio of PMNs to total cells in the BALF and lung MPO activity, an indicator of neutrophil infiltration, 24 h after CLP surgery. PMN/total cell ratio in the BALF (Fig. 3c) and lung MPO activity (Fig. 3d) in CLP-challenged mice were dramatically increased (*P* < 0.05 *versus* the sham group, *n* = 8/group) and were inhibited by AAV-TIPE2, DEX and AAV-TIPE2 + DEX treatment (*P* < 0.05, *n* = 8/group). The lung W/D weight ratio, BALF protein concentration, PMN/total cell ratio in the BALF and lung MPO activity were lower in the combination treatment group than in the AAV-TIPE2 + CLP and CLP + DEX groups.

### Arterial Blood Gas Analysis 24 h After CLP Surgery

**Fig. 4:**
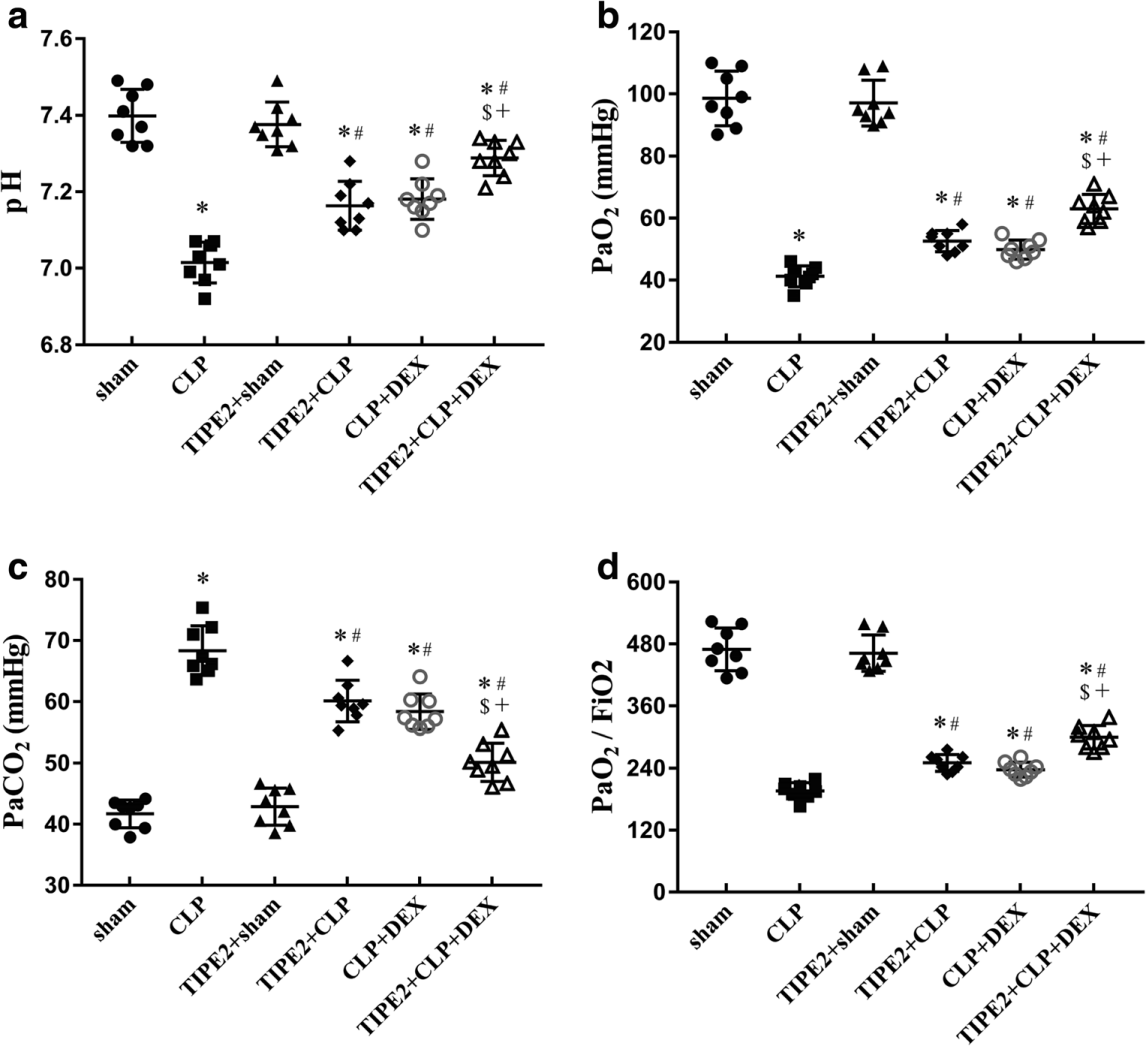
Arterial blood gas analysis at 24 h after CLP surgery. **a** pH. **b** PaO_2_. **c** PaO_2_/FiO_2_. **d** PaCO_2_. The data are presented as means ± SEM. *n* = 8/group, ^*^P < 0.05 *versus* sham group; ^#^*P* < 0.05 *versus* CLP group; ^$^*P* < 0.05 *versus* TIPE2 + CLP group; +*P* < 0.05 *versus* CLP + DEX group.

As shown in Fig. 4, arterial blood gas analysis of mice that received CLP surgery showed significant changes compared with the sham group, with the pH (Fig. 4a), partial pressure of arterial oxygen (PaO_2_) (Fig. 4b) and PaO_2_/FiO_2_ (Fig. 4c) decreasing and the partial pressure of arterial carbon dioxide (PaCO_2_) (Fig. 4d) increasing. The PaO_2_/FiO_2_ of the mice in the CLP group achieved clinical diagnostic criteria for ALI (< 300). Compared with those in the CLP group, the pH, PaO_2_ and PaO_2_/FiO_2_ were increased and the PaCO_2_ was decreased in the TIPE2 + CLP group and CLP + DEX group (*P* < 0.05, *n* = 8/group). Combination treatment showed a more significant effect in improving pulmonary dysfunction. In addition, the PaO_2_/FiO_2_ in the TIPE2 + CLP + DEX group recovered to normal levels.

### Proinflammatory Cytokine Levels in the BALF

**Fig. 5:**
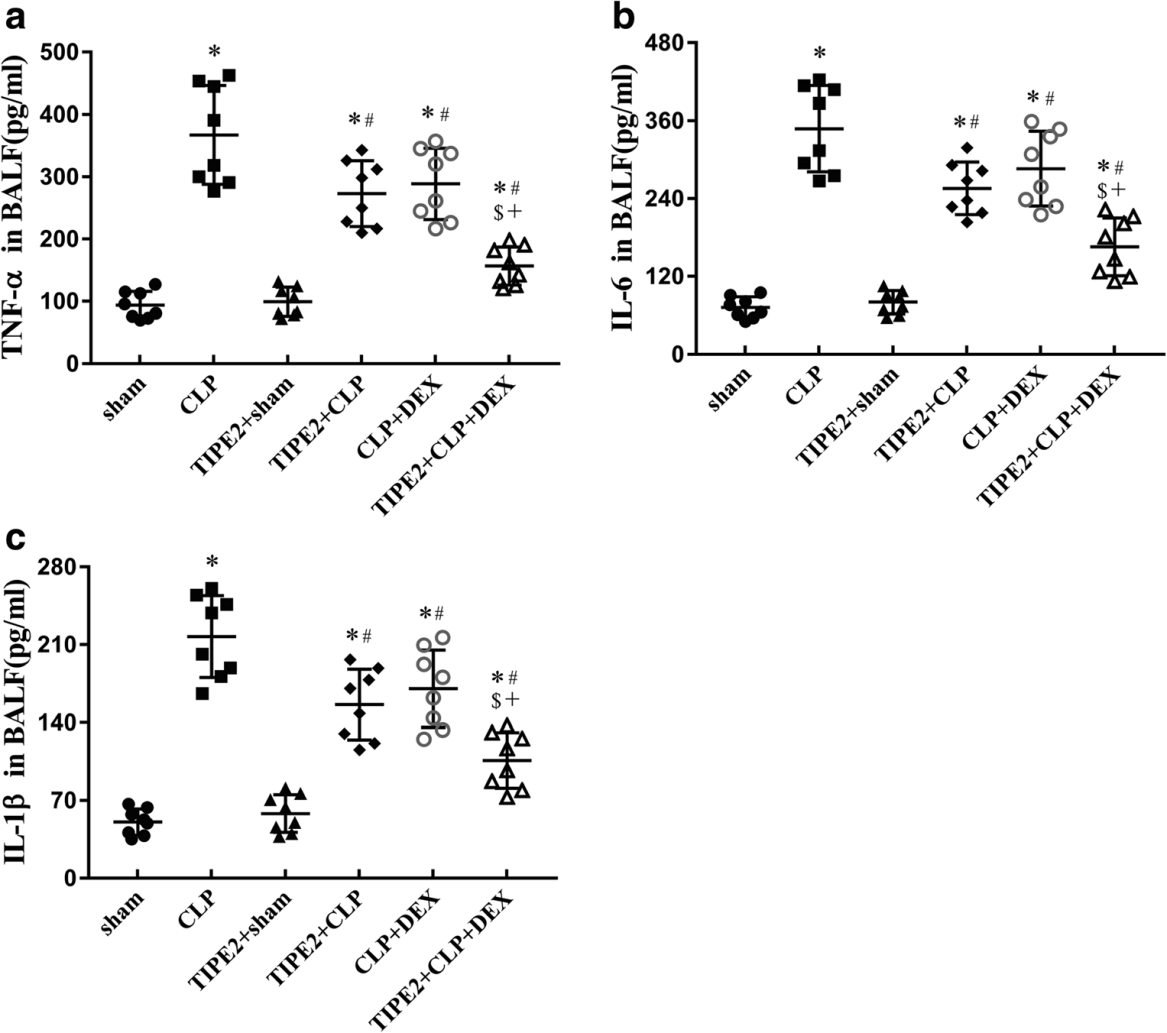
Levels of **a** TNF-α, **b** IL-6 and **c** IL-1β in BALF are presented in mice following sham procedure or CLP surgery at 24 h. The data are presented as means ± SEM, *n* = 8/group, ^*^P < 0.05 *versus* sham group; ^#^*P* < 0.05 *versus* CLP group; ^$^*P* < 0.05 *versus* TIPE2 + CLP group; +*P* < 0.05 *versus* CLP + DEX group.

As depicted in Fig. 5, we found that the levels of the proinflammatory cytokines TNF-α (Fig. 5a), IL-6 (Fig. 5b) and IL-1β (Fig. 5c) in the BALF were significantly increased 24 h after CLP in CLP-challenged mice (*P* < 0.05 *versus* the sham group, *n* = 8/group). AAV-TIPE2 and DEX treatment substantially downregulated the levels of proinflammatory cytokines in the BALF induced by CLP (*P* < 0.05). The cytokine levels in the combination treatment group were lower than those in the AAV-TIPE2 + CLP and CLP + DEX groups (*P* < 0.05).

### The JNK and NF-κB Signalling Pathway and the Protein Expression of Genes Involved in Apoptosis and Lung Injury Induced by CLP

**Fig. 6:**
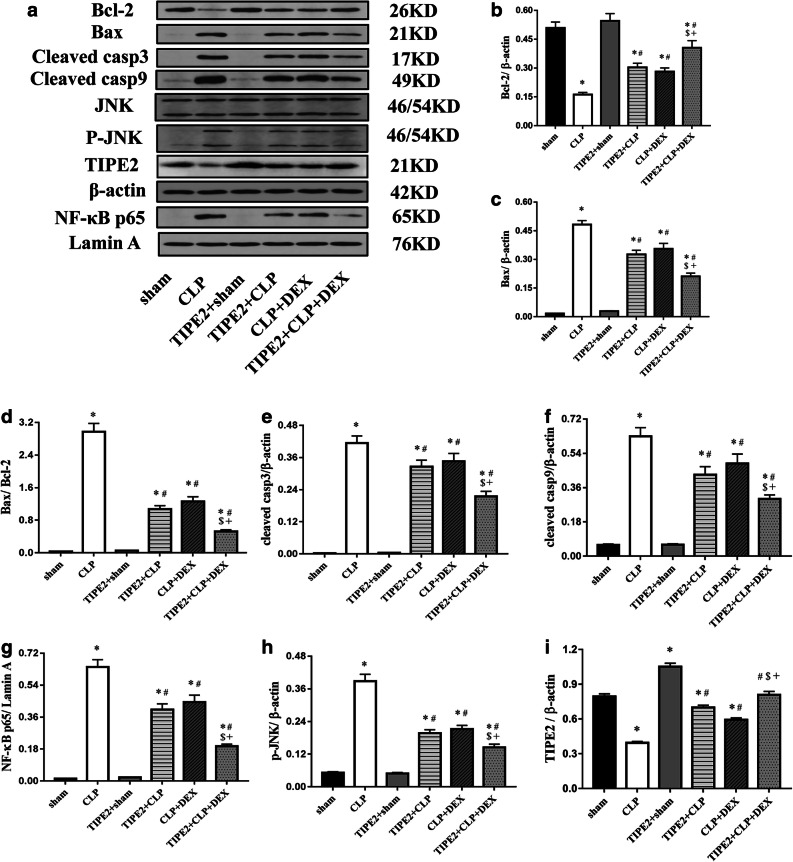
Protein expression of Bcl-2, Bax, cleaved caspase-3, cleaved caspase-9, JNK, p-JNK, TIPE2 and nuclear NF-κB p65 in lung tissues collected at 24h after sham procedure or CLP surgery. **a** Western blotting analysis of protein expression of Bcl-2, Bax, cleaved caspase-3, cleaved caspase-9, JNK, p-JNK, TIPE2 and nuclear NF-κB p65; **b**–**h** The relative ratio of protein expression. The data are presented as means ± SEM, *n* = 8/group, ^*^P < 0.05 *versus* sham group; ^#^*P* < 0.05 *versus* CLP group; ^$^*P* < 0.05 *versus* TIPE2 + CLP group; +*P* < 0.05 *versus* CLP + DEX group.

Previous studies have shown that TIPE2 is a negative regulator of the NF-κB, JNK and p38 MAPK pathways in macrophages [18]. The effect of TIPE2 on the NF-κB and JNK pathways was assessed in mice after CLP challenge. To evaluate the effect of TIPE2 overexpression on lung cell apoptosis in CLP-challenged mice, the protein expression levels of anti-apoptotic (Bcl-2) and proapoptotic (Bax, cleaved caspase-3, cleaved caspase-9) protein in lung tissues were analysed by Western blotting. Mice with CLP-induced ALI exhibited decreased Bcl-2 protein expression and increased Bax, Bax/Bcl-2, cleaved caspase 3 and cleaved caspase 9 protein expression in lung tissue samples compared to that in the sham group, whereas the expression changes in the proteins were reversed by AAV-TIPE2 and DEX treatment. In addition, CLP surgery markedly increased the phosphorylation of JNK (p-JNK) and nuclear NF-κB p65 and decreased TIPE2 expression in the lungs compared with those in the sham group. However, AAV-TIPE2 and DEX treatment suppressed the increase in the expression of nuclear NF-κB p65 and p-JNK induced by LPS. Moreover, the combination group exhibited lower expression of Bax, cleaved caspase 3, cleaved caspase 9, p-JNK and nuclear NF-κB p65 but higher levels of Bcl-2 and TIPE2, compared with those in the AAV-TIPE2 + CLP and CLP + DEX groups (*P* < 0.05) (Fig. 6).

## DISCUSSION

ALI and ARDS are the two main causes of acute lung failure with high morbidity and mortality, and they lack effective therapy strategies [22]. Thus, identifying novel therapeutic treatments for ALI is urgently needed. DEX has been shown to decrease mortality and complications in sepsis patients [23]. In addition, DEX has also been shown to mitigate CLP-stimulated ALI [24]. Several studies have shown that TIPE2 can inhibit NF-κB activation, thereby reducing the generation of proinflammatory mediators and attenuating the sepsis-induced inflammatory response [25]. TIPE2-deficient cells are hyper-responsive to the activation of TLR and TCR signals [18]; in addition, TIPE2-knockout mice, unlike normal WT mice, have been found to exhibit septic shock responses [26].

In the current study, we found that DEX attenuated CLP-induced ALI in mice *via* its anti-inflammatory and anti-apoptotic effects through the upregulation of TIPE2. The key findings are as follows: first, DEX significantly improved the survival rate after CLP challenge; second, DEX significantly mitigated CLP-induced lung injury, as evidenced by the changes in histopathology, the lung W/D weight ratio, the BALF protein concentration and arterial blood gas; third, DEX decreased inflammatory cell infiltration into the lung and pulmonary cell apoptosis; fourth, the levels of proinflammatory cytokines in the BALF in CLP-challenged mice were reduced by DEX treatment; fifth, DEX inhibited CLP-induced lung NF-κB p65 nuclear translocation and JNK phosphorylation but upregulated the protein expression level of TIPE2; finally, DEX prevented lung cell apoptosis by downregulating the expression of pro-apoptotic proteins (Bax, cleaved caspase-3 and cleaved caspase-8) and upregulating the expression of anti-apoptotic protein (Bcl-2) in the lungs of CLP-challenged mice. Similar results were observed in AAV-TIPE2-administered mice, and the combination of AAV-TIPE2 and DEX was more effective in alleviating CLP-induced ALI.

TIPE2 is a critical regulator of immune homeostasis that negatively regulates T cell receptor (TCR) and toll-like receptor (TLR) signalling [18, 25]. TIPE2 deficiency in mice induces foetal inflammatory diseases, and TIPE2 downregulation in humans causes systemic autoimmunity [18, 27]. Moreover, TIPE2, which contains a death effector domain (DED) and is able to inhibit the activities of the apoptotic enzymes caspase3 and caspase8, has also been identified as an apoptosis regulator [28]. Sun et al. demonstrated that the deletion of TIPE2 amplifies JNK and p38 MAPK phosphorylation and NF-κB activation, suggesting that TIPE2 is a negative regulator of these signalling pathways [18].

DEX has been shown to have a beneficial effect on acute lung injury [29]. Hu and colleagues observed that DEX can mitigate CLP-stimulated ALI, and potentially through the reduced the activation of NF-κB and MAPK [24]. However, to date, whether TIPE2 participates in the protective effect of DEX against ALI has not been elucidated.

In the current study, we used CLP to induce sepsis and ALI in mice. CLP-induced sepsis is a commonly used animal model that has high clinical relevance to humans, not only because it is reproducible and comparable to surgical sepsis in patients but also because CLP can induce apoptosis of selected cell types and host immune responses that seem to mimic those in human sepsis [30, 31]. In our study, we found that ALI, which was characterized by pathological changes in lung tissue, increased lung water content, the infiltration of inflammatory cells and pulmonary dysfunction, was present 24 h after CLP. However, AAV-TIPE2 administration and DEX treatment markedly reduced CLP-induced lung injury. More importantly, we found that treatment with AAV-TIPE2 or DEX significantly improved the survival of mice after CLP.

The characteristics of typical ALI are complicated and include the widespread destruction of alveolar epithelium, alveolar haemorrhage, increased lung vascular permeability, PMN infiltration and severe histological damage [32, 33]. Our study showed that AAV-TIPE2 and DEX attenuated the histopathological damage of ALI, including edema, haemorrhage, alveolar wall thickness and PMN recruitment into the lungs of mice after CLP. In ALI, neutrophils significantly contribute to the progression of the host’s inflammatory defence [34, 35]. Neutrophils migrate into the pulmonary alveoli and release proinflammatory cytokines, subsequently damaging pulmonary microvascular endothelial cells [36, 37]. When neutrophils are activated, MPO, which is highly expressed in neutrophils and haemoglobin, can be released into phagosomes and the extracellular matrix. Consequently, the immune activity of MPO may reflect the degree of vascular injury and neutrophil infiltration [38]. The results of our present study showed that the number of PMNs and MPO activity were lower in the AAV-TIPE2 + CLP and CLP + DEX groups than in the CLP group, and the number of PMNs and MPO activity in the combination treatment group were lowest. These findings suggest that TIPE2 overexpression and DEX leads to reduced inflammatory responses in the lungs.

It is believed that NF-κB is a pivotal transcription factor doe the expression of several harmful genes responsible for the pathophysiology of sepsis-induced ALI [39]. When activated, NF-κB p65 translocates into the nucleus where it triggers the transcription of inflammatory cytokines, such as TNF-α, IL-6 and IL-1β [40]. The inactivation of NF-κB p65 inhibits inflammation-induced inflammatory cell infiltration, edema and proinflammatory cytokine production in the lungs [41]. In the current study, both TIPE2 overexpression and DEX treatment significantly inhibited the levels of TNF-α, IL-6 and IL-1β in the BALF induced by CLP and concomitantly decreased NF-κB activation, as evidenced by decreased levels of nuclear NF-κB p65 in the lung. Therefore, the inhibitory effect of TIPE2 overexpression and DEX on CLP-increased levels of proinflammatory cytokines may be ascribed to the suppression of NF-κB activation.

Pulmonary cell apoptosis also plays a critical role in the pathogenesis of ALI [42]. Evidence implicates increased epithelial/endothelial cell apoptosis in the pathogenesis of ALI. In addition, several studies have shown that ALI is associated with increased cell death in critically ill patients [43, 44]. Caspase-3 plays a key role in apoptotic cell death [45], and our present study revealed a significant increase in the level of cleaved caspase-3 after CLP surgery, indicating increased cell apoptosis in the lungs. MAPK family molecules are important regulators involved in the production of cytokines and mediators associated with the pathogenesis of inflammatory processes [46]. JNK, a member of the MAPK family, has been implicated in many cellular events, including apoptosis [47]. A variety of stress signals, such as cytokines and inflammation, can trigger JNK phosphorylation, which subsequently mediates the phosphorylation of anti-apoptotic proteins (Bcl-2/Bcl-xL) and upregulates pro-apoptotic Bax [48]. When the Bax to Bcl-2 ratio elevates, the mitochondrial membrane potential changes, causing the release of cytochrome C from mitochondria into the cytosol [49], resulting in the activation of caspase 9 and then caspase 3 to induce apoptosis [50]. In the current study, we found that AAV-TIPE2 and DEX remarkably inhibited the expression of pro-apoptotic proteins (Bax, cleaved caspase-9 and cleaved caspase-3) and JNK phosphorylation induced by CLP and restored the expression of the anti-apoptotic protein Bcl-2. These findings suggest that the inhibitory effect of TIPE2 overexpression and DEX on CLP-induced cell apoptosis may be attributed to the suppression of JNK activation. Moreover, Western blotting results revealed that the TIPE2 protein expression level was significantly decreased after CLP challenge; however, DEX treatment increased the TIPE2 level compared with that in the CLP group.

A limitation of our current study is that it focused on the first 24 h after CLP, and the question of whether DEX or AAV-TIPE2 exert similar beneficial effects at other times remains to be explored. As severe sepsis occurs 24 h after CLP surgery, we chose this time point to assess ALI, but further studies may need to be performed at other times.

In conclusion, our study demonstrated that TIPE2 expression was significantly decreased in lung tissues in ALI mice after CLP challenge. Moreover, both adeno-associated virus-mediated TIPE2 overexpression and DEX treatment remarkably inhibited inflammation and cell apoptosis induced by CLP. We have also provided evidence that the anti-inflammatory and anti-apoptotic effects of DEX might at least partly involve the upregulation of TIPE2 and the inhibition of the NF-κB and JNK signalling pathways. Therefore, our results indicate that DEX might be useful as a potential therapeutic target for sepsis-induced ALI.

## Abbreviations

ALI: acute lung injury
ARDS: acute respiratory distress syndrome
CLP: caecal ligation-perforation
BALF: bronchoalveolar lavage fluid
ELISA: enzyme-linked immunosorbent assay
MPO: myeloperoxidase
PBS: phosphate-buffered saline
PMNs: polymorphonuclear neutrophils
TUNEL: deoxynucleotidyl transferase dUTP nick end labelling
W/D: wet-to-dry
TIPE2: tumour necrosis factor-α-induced protein 8-like 2
DEX: dexmedetomidine
Bax: Bcl-2-associated X
Bcl-2: B cell lymphoma 2

## References

[1] Walkey AJ, Kirkpatrick AR, Summer RS (2015). Systemic inflammatory response syndrome criteria for severe Sepsis. The New England Journal of Medicine.

[2] Khan MM, Yang WL, Brenner M, Bolognese AC, Wang P (2017). Cold- inducible RNA-binding protein (CIRP) causes sepsis-associated acute lung injury via induction of endoplasmic reticulum stress. Scientific Reports.

[3] Li C, Yang D, Cao X, Wang F, Jiang H, Guo H (2016). LFG-500, a newly synthesized flavonoid, attenuates lipopolysaccharide-induced acute lung injury and inflammation in mice. Biochemical Pharmacology.

[4] Zhao Z, Tang X, Zhao X, Zhang M, Zhang W, Hou S (2014). Tylvalosin exhibits anti-inflammatory property and attenuates acute lung injury in different models possibly through suppression of NF-kappaB activation. Biochemical Pharmacology.

[5] Matthay MA, Zimmerman GA (2005). Acute lung injury and the acute respiratory distress syndrome: Four decades of inquiry into pathogenesis and rational management. American Journal of Respiratory Cell and Molecular Biology.

[6] Singh G, Gladdy G, Chandy TT, Sen N (2014). Incidence and outcome of acute lung injury and acute respiratory distress syndrome in the surgical intensive care unit. Indian J Crit Care Med.

[7] Z'graggen BR, Tornic J, Müller-Edenborn B, Reyes L, Booy C, Beck-Schimmer B (2010). Acute lung injury: Apoptosis in effector and target cells of the upper and lower airway compartment. Clinical and Experimental Immunology.

[8] Hotchkiss RS, Swanson PE, Freeman BD, Tinsley KW, Cobb JP, Matuschak GM (1999). Apoptotic cell death in patients with sepsis, shock, and multiple organ dysfunction. Critical Care Medicine.

[9] Davis RJ (2000). Signal transduction by the JNK group of MAP kinases. Cell.

[10] Lin A (2003). Activation of the JNK signaling pathway: Breaking the brake on apoptosis. Bioessays.

[11] Cho JS, Shim JK, Soh S, Kim MK, Kwak YL (2016). Perioperative dexmedetomidine reduces the incidence and severity of acute kidney injury following valvular heart surgery. Kidney International.

[12] Taniguchi T, Kidani Y, Kanakura H, Takemoto Y, Yamamoto K (2004). Effects of dexmedetomidine on mortality rate and inflammatory responses to endotoxin-induced shock in rats. Critical Care Medicine.

[13] Sanders RD, Sun P, Patel S, Li M, Maze M, Ma D (2010). Dexmedetomidine provides cortical neuroprotection: Impact on anaesthetic-induced neuroapoptosis in the rat developing brain. Acta Anaesthesiologica Scandinavica.

[14] Zhang Q, Wu D, Yang Y, Liu T, Liu H (2017). Dexmedetomidine alleviates Hyperoxia-induced acute lung injury via inhibiting NLRP3 Inflammasome activation. Cellular Physiology and Biochemistry.

[15] Zhang Y, Jia S, Gao T, Zhang R, Liu Z, Wang Y (2018). Dexmedetomidine mitigate acute lung injury by inhibiting IL-17-induced inflammatory reaction. Immunobiology.

[16] Géloën A, Pichot C, Leroy S, Julien C, Ghignone M, May CN (2015). Pressor response to noradrenaline in the setting of septic shock: Anything new under the Sun-Dexmedetomidine, clonidine? A Minireview. BioMed Research International.

[17] Kumar D, Gokhale P, Broustas C, Chakravarty D, Ahmad I, Kasid U (2004). Expression of SCC-S2, an antiapoptotic molecule, correlates with enhanced proliferation and tumorigenicity of MDA-MB 435 cells. Oncogene.

[18] Sun H, Gong S, Carmody RJ, Hilliard A, Li L, Sun J (2008). TIPE2, a negative regulator of innate and adaptive immunity that maintains immune homeostasis. Cell.

[19] Siempos II, Lam HC, Ding Y, Choi ME, Choi AM, Ryter SW (2014). Cecal ligation and puncture-induced sepsis as a model to study autophagy in mice. Journal of Visualized Experiments.

[20] Jeremias IC, Victorino VJ, Barbeiro HV, Kubo SA, Prado CM, Lima TM (2016). The role of acetylcholine in the inflammatory response in animals surviving Sepsis induced by Cecal ligation and puncture. Molecular Neurobiology.

[21] Su X, Wang L, Song Y, Bai C (2004). Inhibition of inflammatory responses by ambroxol, a mucolytic agent, in a murine model of acute lung injury induced by lipopolysaccharide. Intensive Care Medicine.

[22] Johnson ER, Matthay MA (2010). Acute lung injury: Epidemiology, pathogenesis, and treatment. Journal of Aerosol Medicine and Pulmonary Drug Delivery.

[23] Pandharipande PP, Sanders RD, Girard TD, McGrane S, Thompson JL, Shintani AK (2010). Effect of dexmedetomidine versus lorazepam on outcome in patients with sepsis: An a priori-designed analysis of the MENDS randomized controlled trial. Critical Care.

[24] Hu H, Shi D, Hu C, Yuan X, Zhang J, Sun H (2017). Dexmedetomidine mitigates CLP-stimulated acute lung injury via restraining the RAGE pathway. American Journal of Translational Research.

[25] Freundt EC, Bidere N, Lenardo MJ (2008). A different TIPE of immune homeostasis. Cell.

[26] Xi W, Hu Y, Liu Y, Zhang J, Wang L, Lou Y (2011). Roles of TIPE2 in hepatitis B virus-induced hepatic inflammation in humans and mice. Molecular Immunology.

[27] Li D, Song L, Fan Y, Li X, Li Y, Chen J (2009). Down-regulation of TIPE2 mRNA expression in peripheral blood mononuclear cells from patients with systemic lupus erythematosus. Clinical Immunology.

[28] Kumar D, Whiteside TL, Kasid U (2000). Identification of a novel tumor necrosis factor-alpha-inducible gene, SCC-S2, containing the consensus sequence of a death effector domain of fas-associated death domain-like interleukin- 1beta-converting enzyme-inhibitory protein. The Journal of Biological Chemistry.

[29] Yan X, Cheng X, Zhou L, He X, Zheng W, Chen H (2017). Dexmedetomidine alleviates lipopolysaccharide-induced lung injury in Wistar rats. Oncotarget.

[30] Buras JA, Holzmann B, Sitkovsky M (2005). Animal models of sepsis: Setting the stage. Nature Reviews. Drug Discovery.

[31] Hubbard WJ, Choudhry M, Schwacha MG, Kerby JD, Rue LW, Bland KI (2005). Cecal ligation and puncture. Shock.

[32] Abraham E, Singer M (2007). Mechanisms of sepsis-induced organ dysfunction. Critical Care Medicine.

[33] Matthay MA, Zemans RL (2011). The acute respiratory distress syndrome: Pathogenesis and treatment. Annual Review of Pathology.

[34] Brown KA, Brain SD, Pearson JD, Edgeworth JD, Lewis SM, Treacher DF (2006). Neutrophils in development of multiple organ failure in sepsis. Lancet.

[35] Yang KY, Arcaroli JJ, Abraham E (2003). Early alterations in neutrophil activation are associated with outcome in acute lung injury. American Journal of Respiratory and Critical Care Medicine.

[36] Zhang HX, Liu SJ, Tang XL, Duan GL, Ni X, Zhu XY (2016). H2S attenuates LPS-induced acute lung injury by reducing oxidative/Nitrative stress and inflammation. Cellular Physiology and Biochemistry.

[37] Grommes J, Soehnlein O (2011). Contribution of neutrophils to acute lung injury. Molecular Medicine.

[38] Hirano Y, Aziz M, Yang WL, Wang Z, Zhou M, Ochani M (2015). Neutralization of osteopontin attenuates neutrophil migration in sepsis-induced acute lung injury. Critical Care.

[39] Liu SF, Malik AB (2006). NF-kappa B activation as a pathological mechanism of septic shock and inflammation. American Journal of Physiology. Lung Cellular and Molecular Physiology.

[40] Wang H, Wang L, Li NL, Li JT, Yu F, Zhao YL (2014). Subanesthetic isoflurane reduces zymosan-induced inflammation in murine Kupffer cells by inhibiting ROS-activated p38 MAPK/NF-kappaB signaling. Oxidative Medicine and Cellular Longevity.

[41] Blackwell TS, Blackwell TR, Holden EP, Christman BW, J.W. (1996). Christman. *In vivo* antioxidant treatment suppresses nuclear factor-kappa B activation and neutrophilic lung inflammation. Journal of Immunology.

[42] Galani V, Tatsaki E, Bai M, Kitsoulis P, Lekka M, Nakos G (2010). The role of apoptosis in the pathophysiology of acute respiratory distress syndrome (ARDS): An up-to-date cell-specific review. Pathology, Research and Practice.

[43] Chopra M, Reuben JS, Sharma AC (2009). Acute lung injury:Apoptosis and signaling mechanisms. Experimental Biology and Medicine (Maywood, N.J.).

[44] Perl M, Chung CS, Perl U, Thakkar R, Lomas-Neira J, Ayala A (2010). Therapeutic accessibility of caspase-mediated cell death as a key pathomechanism in indirect acute lung injury. Critical Care Medicine.

[45] Porter AG, Janicke RU (1999). Emerging roles of caspase-3 in apoptosis. Cell Death and Differentiation.

[46] Lee IT, Yang CM (2013). Inflammatory signalings involved in airway and pulmonary diseases. Mediators of Inflammation.

[47] Kyriakis JM, Banerjee P, Nikolakaki E, Dai T, Rubie EA, Ahmad MF (1994). The stress-activated protein kinase subfamily of c-Jun kinases. Nature.

[48] Sui X, Kong N, Ye L, Han W, Zhou J, Zhang Q (2014). p38 and JNK MAPK pathways control the balance of apoptosis and autophagy in response to chemotherapeutic agents. Cancer Letters.

[49] Kluck RM, Bossy-Wetzel E, Green DR, Newmeyer DD (1997). The release of cytochrome c from mitochondria: A primary site for Bcl-2 regulation of apoptosis. Science.

[50] Li B, Zeng M, He W, Huang X, Luo L, Zhang H (2015). Ghrelin protects alveolar macrophages against lipopolysaccharide-induced apoptosis through growth hormone secretagogue receptor 1a-dependent c-Jun N-terminal kinase and Wnt/beta-catenin signaling and suppresses lung inflammation. Endocrinology.

